# Coordinated Response to Reports of Possible Anthrax Contamination, Idaho, 2001

**DOI:** 10.3201/eid0810.020390

**Published:** 2002-10

**Authors:** Leslie Tengelsen, Richard Hudson, Shana Barnes, Christine Hahn

**Affiliations:** * Idaho Department of Health and Welfare, Boise, Idaho, USA; † State Emergency Management Systems, Meridian, Idaho, USA

**Keywords:** anthrax, bioterrorism, Idaho

## Abstract

In 2001, the intentional release of anthrax spores in the eastern United States increased concern about exposure to anthrax nationwide, and residents of Idaho sought assistance. Response from state and local agencies was required, increasing the strain on epidemiologists, laboratorians, and communications personnel. In late 2001, Idaho’s public health communications system handled 133 calls about suspicious powders. For each call, a multiagency bridge call was established, and participants (public health officials, epidemiologists, police, Federal Bureau of Investigation personnel, hazardous materials officials, and others) determined which samples would be tested by the state public health laboratory. A triage system for calls helped relieve the burden on public safety and health systems.

After the intentional spread of anthrax spores in 2001, states without anthrax cases were nonetheless affected by the outbreak. Idaho recorded a sharp rise in emergency calls, and the response requirements for traditional first responders, public health officials, epidemiologists, laboratorians, and communications personnel increased. Before the outbreak, public health officials and first responders had little experience in jointly managing health-related issues. New response protocols and functional interagency relationships needed to be developed rapidly. Responders were faced with new scenarios and an increased call volume. In addition, safe handling protocols were needed for managing potential anthrax cases and handling clinical samples. The response and distribution of timely, accurate information between local, state, and federal public health partners, first responders, the health-care community, and the general public were crucial. Through this experience, procedures have been streamlined for a more effective response.

## Notification and Initial Response to Possible Anthrax Exposures

Anecdotal information suggests that all states had to respond to public inquiries about powdery substances found in the mail or public areas. Despite being removed geographically from anthrax cases and contaminated sites, Idaho was no exception. The state uses a centralized State Emergency Medical Services Communications Center (StateComm), which receives emergency calls in areas that lack 911 services and provides the emergency communication system for and between all state agencies. This center was established in 1974 through a Robert Wood Johnson Foundation grant to enhance rural Emergency Management System communications services but has expanded over the last 10 years to include public health inquiries. StateComm, which is part of the Idaho Department of Health and Welfare, operates 22 remote mountaintop transmitter sites connected by microwave links to a central location. StateComm staff dispatch regional hazardous materials (hazmat) teams, page public health officials, and provide bridge call services; up to 48 ports are available for a single bridge call.

**Figure 1:**
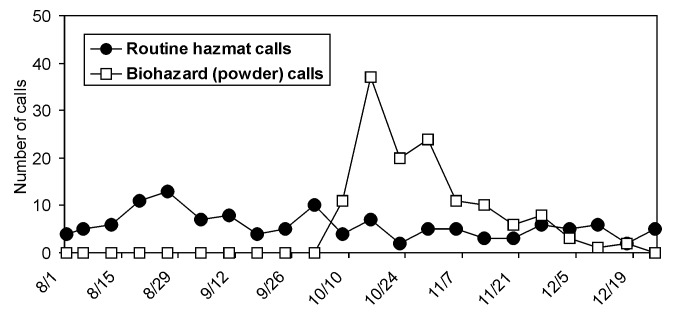
Calls received by the Idaho State Communications Center from August 1, 2001 to December 31, 2001, are shown by category: routine hazardous materials calls and biohazard (suspicious powder calls).

From August 1 to October 7, 2001, StateComm received 73 routine hazmat calls and no biohazard calls, which was a typical calling pattern for the hotline (Figure 1). However, from October 8 to December 31, 2001, StateComm received 53 routine hazmat calls and 133 biohazard calls; all biohazard calls were related to suspicious powders. Most of the biohazard calls were made by local law enforcement, who were on-scene incident commanders following state hazmat response protocols during powder investigations. StateComm staff then convened emergency bridge calls for each biohazard call and used state hazmat protocols to determine who should participate in the call. Public health, law enforcement (including Federal Bureau of Investigation [FBI]), hazmat, and other officials routinely participated in these calls and discussed how to respond to possible anthrax exposures. All powder-related incidents were treated as potential criminal acts, and all samples were maintained as evidence to ensure a standardized response. For each call, participants asked the incident commander if a written threat was present and who was the apparent target. If an envelope or package had a return address, the on-scene incident commander contacted the sender to verify that he or she sent the item and to identify its contents. The threat level was then assessed based on suspicious package guidelines (1) and other requirements listed previously.

During the first days of calls, emergency bridge call participants agreed that no samples would be sent to the state public health laboratory for testing until approved by call participants. The state public health laboratory was notified when samples were routed to them. Persons who may have been exposed to anthrax were informed by the on-scene incident commander that results would be available within 48 h, that antibiotics were not recommended pending test results, and that they were free to consult with their medical provider. With this protocol, only 50 (37.6%) of the biohazard calls yielded items for testing by the state Bureau of Laboratories. All test results from the laboratory were reported directly to StateComm, which then notified the on-scene incident commander of the results.

## Laboratory Testing

The state public health laboratory in Boise is the only laboratory in Idaho that accepts environmental samples for anthrax testing. All 50 suspect exposure incidents, as determined by a multiagency bridge call, were given numeric identifiers by StateComm and linked to powder samples being routed to the state public health laboratory. Transportation across Idaho was facilitated by a state police escort to maintain the chain of custody. The state public health laboratory established an on-site chain of custody protocol with local, state, or FBI law enforcement officials before the microbiologic evaluation of any item. Thirty incidents yielded postmarked items for testing (letters, envelopes, and packages). Seven incidents yielded swabs or vials of powder for testing. Miscellaneous objects received for testing included clothing, a mailbox, a handheld vacuum, a pillbox, a toy, a dollar bill, and a crate. Three of the letters contained threats, which necessitated FBI involvement. Objects with possible contamination were evaluated for spores with the spore stain (Malachite green) by wet-mount-phase microscopy and were cultured for *Bacillus anthracis* under modified biosafety level-3 conditions (2). Although some objects contained *Bacillus* species, all were negative for *Bacillus anthracis* by gamma-phage testing. A turnaround time of 24 h or less was generally maintained for presumptive determinations, and StateComm was alerted immediately of presumptive negative test results. A final culture-negative determination was made 48 h after receipt of the sample. Laboratorians followed protocols provided by the Centers for Disease Control and Prevention Laboratory Response Network. That the staff of three microbiologists was not enough to handle the dramatic increase in workload was soon evident. Therefore, 11 additional laboratorians were trained in test procedures, and the staff was grouped into two-person teams to provide around-the-clock coverage. Facility biosecurity was increased, with locked entries, a sign-in desk, and guest badges.

## Health-Care Outreach

Idaho has documented rare, naturally occurring cases of anthrax. The last human case of cutaneous anthrax occurred in 1964, and the last documented animal case occurred in a cow in 1984. The state epidemiology staff developed two sets of public health guidelines for health-care providers, which included information about the epidemiology of naturally occurring anthrax in Idaho, the features of the current outbreak of anthrax (1,3,4), and the possible risk to postal workers. The guidelines also included information about the availability of in-state testing and state and local public health contacts. These guidelines were faxed to the seven district health departments, which in turn faxed them to health-care providers, emergency rooms, and infection control practitioners, following the Health Alert Network system protocols. The guidelines were also placed on the state health department Web site and faxed or mailed to providers, media, and citizens who requested anthrax information.

During October 2001, local physicians contacted the state epidemiology office for assistance in evaluating and treating 12 possible anthrax cases: 11 persons with possible inhalational anthrax (6 [54.5%] were postal workers) and 1 person with possible cutaneous anthrax. All human samples were negative for anthrax. To better understand what syndromic signs and symptoms created suspicion in health-care providers, we reviewed 9 of 12 suspected anthrax cases (Table). Information was gathered and compared with the first 10 confirmed inhalational anthrax cases in the United States (5). Occupational risk played a key role in suspicion of pulmonary anthrax infection; however, the symptoms of the suspected anthrax cases varied greatly from those of confirmed anthrax cases. These findings were included in follow-up information sent to health-care providers.

**Table:** Clinical comparison of confirmed versus suspected inhalational anthrax cases

Characteristics	^a^Confirmed cases, n=10 (%)	Idaho suspected cases, n=9^b^ (%)
Postal worker or mail sorter	8 (80)	6/11^c^ (54)
Fever/chills	10 (100)	2 (22)
Fatigue/malaise	10 (100)	8 (89)
Sweats	7 (70)	2 (22)
Cough	9 (90)	7 (78)
Nausea or vomiting	9 (90)	2 (22)
Dyspnea	8 (80)	3 (33)
Rhinorrhea	1 (10)	4 (44)

^a^Cases confirmed by Centers for Disease Control and Prevention. ^b^Nine of the suspected inhalational cases had charts available for review. ^c^Eleven suspected inhalational anthrax cases and one suspected cutaneous case.

## Discussion

Reviewing the problems encountered in Idaho and how they were addressed may improve the public health response in all states. A centralized communications center is critical for reducing the impact of a large-scale outbreak on a public health emergency response system and for providing timely and consistent response to citizens. In Idaho, the preexisting communications system enabled communication between multiple agencies. The rapid development of triage protocols is important for consistent response to a crisis. While basic response protocols must be outlined for each event, a rapid mechanism for protocol development and agreement by participants must be part of any flexible response plan. Local health departments should be included in biohazard response protocols to minimize confusion during the management and follow-up of each public health event. Initially, StateComm calls included only state health department officials because local health officials did not carry pagers. Local health officials in Idaho are now equipped with pagers and are part of the response protocol. Immediate reporting of laboratory test results to a central communications center reduces the burden on laboratory staff. The volume of callers seeking results was decreased because health and law officials were aware that results could be obtained from the communications center directly. Extra effort and time attempting to reach first responders, citizens, and health officials with test results were also eliminated.

In responding to suspected bioterrorist events, treating each event as a possible crime requires cooperation and planning. Transport of samples by law enforcement required cooperation with multiple county law enforcement officials. Alternative transportation plans would have been useful in Idaho should a local law enforcement agency have refused to transport a specimen. In addition, the establishment of a proper chain of custody and proper packaging procedures would have allowed more streamlined processing of samples for both laboratory safety and chain-of-custody requirements. Education of state communications personnel in communicable disease topics, such as anthrax, is required if communications personnel are used to initiate and coordinate response protocols to biohazard events. A basic understanding of the terminology and the general principles of epidemiologic response would minimize the chance that a potentially serious situation is overlooked. Trust between the decision-makers in multiple state, local, and federal agencies is essential for coordinating responses effectively. In small states, fewer people are usually involved in each response, and the same participants tend to be on each call, simplifying coordination. Planning meetings with other responding agencies are essential in order to establish protocols and to foster trust.

States with small, rural populations often have fewer resources to deal with the increasing stress on their emergency response systems. Despite the lack of anthrax infections in the western United States during the fall of 2001, citizens in Idaho were fearful of being exposed to anthrax, and the public health emergency response system was tested. A well-coordinated response was required from agencies with little experience in working together. Idaho was fortunate to have a statewide communications network in place; however, even with this response system, modifications were required to ensure smooth relationships between first responders and public health officials.
